# The impact of psychological factors on outcome after salvage surgery for wrist osteoarthritis

**DOI:** 10.1177/17531934221104603

**Published:** 2022-06-14

**Authors:** Elin M. Swärd, Gunilla Brodda-Jansen, Thorsten U. Schriever, Mikael Andersson-Franko, Maria K. Wilcke

**Affiliations:** 1 Karolinska Institutet, Department of Clinical Science and Education, Södersjukhuset, Stockholm, Sweden; 2 Department of Hand Surgery Södersjukhuset, Stockholm, Sweden; 3 Karolinska Institutet, Department of Clinical Sciences, Division of Rehabilitation Medicine, Danderyd University Hospital, Stockholm, Sweden

**Keywords:** Wrist, osteoarthritis, anxiety, pain catastrophizing, psychological, outcome, surgery

## Abstract

This prospective longitudinal study of 80 patients analysed the effect of preoperative pain catastrophizing, anxiety, depression and sense of coherence on the Disabilities of the Arm, Shoulder and Hand, Patient-Rated Wrist Evaluation, quality of life, grip strength and range of motion during the first year after salvage surgery for wrist osteoarthritis. Generalized estimating equations were used to analyse the effect of the psychological factors on the outcome variables. Pain catastrophizing or a tendency for anxiety preoperatively had a strong negative impact on postoperative Disabilities of the Arm, Shoulder and Hand and Patient-Rated Wrist Evaluation. Anxiety also predicted a lower postoperative quality of life, whereas pain catastrophizing had a negative impact on grip strength. Sense of coherence did not influence the outcome.

**Level of evidence:** II

## Introduction

Patient-reported function and pain relief after salvage surgery for wrist osteoarthritis varies, and patients with similar diagnoses and degree of osteoarthritis who receive the same type of surgery and rehabilitation may rate their outcome differently. In recent years, there has been increasing interest in the impact of psychological factors on the post-surgical outcome. Anxiety, depression and pain catastrophizing have been reported to predict poor outcome, persisting pain and delayed recovery after surgery in a wide variety of chronic and traumatic medical conditions in the upper extremity (Crijns et al., 2019; Dekker et al., 2016; Egloff et al., 2017; Mosegaard et al., 2020; Ryan et al., 2022; Vranceanu et al., 2010; Yeoh et al., 2016). Sense of coherence (SOC), the ability to cope with stressful situations or challenges, has also been reported to influence outcomes after hand injuries (Carlsson and Dahlin, 2014; Cederlund et al., 2010; Rosberg, 2014).

The influence of psychological status on the outcome after salvage procedures for wrist osteoarthritis has not been investigated. The primary aim of this study was to analyse whether pain catastrophizing, anxiety, depression and SOC has any impact on the result in terms of patient-reported outcome measures (PROMs), grip strength and range of motion (ROM) after salvage procedures for wrist osteoarthritis during the first year after surgery. We hypothesized that depression, anxiety and pain catastrophizing would have a negative effect on the Disability of the Arm, Shoulder and Hand (DASH) score, Patient-Rated Wrist Evaluation (PRWE), quality of life, grip strength and ROM, whereas a high SOC would have a positive impact. The secondary aim was to investigate whether the psychological scores change during the first year after surgery. The null hypothesis was that the pre- and postoperative psychological scores would be equal.

## Methods

### Study design and study population

A prospective longitudinal 1-year follow-up study of 80 patients surgically treated by salvage procedures for painful wrist osteoarthritis was conducted at a specialized hand surgery unit between January 2018 and October 2020. The study population consisted of patients that were enrolled in either a prospective longitudinal study on the outcome after partial wrist denervation (*n *= 49) or a prospective randomization between two methods of midcarpal fusion (*n *= 31). The inclusion criteria were chronic (≥6 months) symptomatic wrist osteoarthritis caused by scapholunate advanced collapse (SLAC) or scaphoid nonunion advanced collapse (SNAC), osteoarthritis after distal radial fracture or secondary to Kienböck’s disease. Only patients that had tried nonsurgical management, such as bracing, corticosteroid injections, oral analgesics and/or physiotherapy, were included. The exclusion criteria were age <18 years and inability to cooperate with the follow-up protocol (owing to language difficulties, severe psychiatric disorder, cognitive impairment or drug addiction). All operations were carried out by surgeons with level three expertise (Tang and Giddins, 2016).

### Outcome variables

The primary outcome variable was change in DASH score (Hudak et al., 1996) (0–100 points; higher score indicating worse function) measured before surgery and 6 and 12 months after operation.

Secondary outcome variables were changes in PRWE (MacDermid et al., 1998) (0–100 points; higher score indicating worse function), EuroQol-5D-3L (EQ5D-3L)) (Brooks, 1996) (0–1 points; higher score indicating better quality of life) measured at the same timepoints as above, and objective function (grip strength and extension–flexion arc of the affected hand) measured by a physiotherapist or hand surgeon before surgery and 12 months after operation. Grip strength was measured with a hydraulic hand dynamometer (BL5001; B&L Engineering®, Santa Ana, CA, USA) and recorded as the mean of three attempts at maximal grip; ROM was measured using a goniometer with 5° intervals. The PROMs were sent to research participants by mail. Data on age, sex, dominant hand and occupation were collected preoperatively.

### Psychological scores

The self-administered psychological questionnaires were measured preoperatively, and at 6 and 12 months postoperatively.

The Pain Catastrophizing Scale (PCS) (Sullivan et al., 1995) is a 13-item questionnaire divided into three subscales that analyses different aspects of pain: rumination (impossible to stop thinking about pain); magnification (worrying that something bad may happen); and helplessness (pain intensity cannot be reduced). The total score ranges from 0 to 52 and previous research indicates that a PCS ≥ 30 represents a clinically relevant pain catastrophizing (Sullivan et al., 1995). PCS ≥ 30 was therefore used as cut-off.

The Hospital Anxiety and Depression Scale (HADS) (Zigmond and Snaith, 1983) comprises 14 items, of which seven relate to anxiety and seven to depressive symptoms. The total score for the anxiety and depression subscales varies from 0 to 21. HADS does not provide a definitive diagnosis, but a score of 8–10 on the respective subscale suggest that a depressive or anxiety disorder may exist and ≥11 indicates a probable depressive or anxiety disorder. HADS ≥ 8 was used as cut-off in this study.

The sense of coherence (SOC-13) (Antonovsky, 1993) reflects a lasting feeling of inner confidence and an experience of life as being manageable, meaningful and comprehensible. The SOC-13 is a 13-item score that measure the coping capacity of people to handle stressful situations. The total score ranges from 13 to 91 points. The higher the score, the stronger the SOC. No cut-off points have been determined for SOC. The median SOC was used to dichotomize into low and high SOC in the study population.

### Statistics

To detect a difference of 10 points in DASH score, which is considered the minimal clinically important difference (MCID) (Sorensen et al., 2013), between dichotomized groups, a total sample size of 73 patients was required (power 80%; *p *= 0.05). SD was estimated to 15. Eighty patients were included to account for some loss to follow-up.

Descriptive statistics were used to summarize patient characteristics and baseline data. DASH, PRWE, EQ5D, SOC, PCS and HADS scores were reported as median (IQR) and continuous variables as mean (SD). To analyse the repeated measurements, we used two separate generalized estimating equations (GEE) with a robust estimator covariance matrix, autoregressive (AR1) working correlation matrix and a linear model for all variables. In the first model DASH, PRWE, EQ5D, grip strength and ROM were dependent variables with time, sex, age, type of surgery and preoperative PCS ≥ 30, HADS ≥ 8 and SOC ≥ 73 included as possible predictors. In the second model PCS, HADS and SOC were dependent variables with time, age, sex and type of surgery as covariates. The changes in outcome variables over time and the effects of the predictors are presented as beta coefficient (β) with *p*-value, where β reflects the expected population mean change of the outcome variables between the preoperative assessment and the assessments 6 and 12 months after operation. Significance was set at *p *≤ 0.05.

## Results

One patient was lost to follow-up 3 months after operation owing to the development of severe psychiatric problems, leaving 79 patients for analysis. Twelve-month data were missing in two patients from the denervation group, who dropped out after 6 months since they required additional wrist surgery for persisting symptoms. Owing to the COVID-19 pandemic, four patients declined clinical assessment and therefore objective physical variables are missing for those patients at one timepoint. Twelve-month physical data are also missing in one patient who moved during the study period. Completeness of the DASH scores was 97%, PRWE 97%, EQ5D 97%, SOC 95%, HADS 96% and PCS 97%, respectively.

The types of wrist osteoarthritis and demographic data are described in Table 1.

**Table 1.** Demographics.

	Type of surgery		Total *n*
	Partial wrist fusion *n*	Partial denervation *n*	
Number of patients (*n*)	31	48	79
Age in years (SD)	61 (10)	60 (15)	60 (14)
Diagnosis
SLAC1	2	4	6
SLAC2	9	13	22
SLAC3	16	17	33
SNAC1	3	3	6
SNAC2	1	1	2
SNAC3	0	4	4
Kienböck’s disease	0	2	2
Arthrosis after distal radial fracture	0	4	4
Gender			
Male	27	33	60
Female	4	15	19
Type of work			
Manual	18	25	43
Office	7	5	12
Retired	6	19	25
Operated dominant hand			
Yes	21	34	55
No	10	14	24

SLAC: scapholunate advanced collapse; SNAC: scaphoid nonunion advanced collapse.

The mean and median values of the outcome variables preoperatively and 6 and 12 months postoperatively are presented in Table 2.

**Table 2.** Patient-reported outcome measures (PROMs), psychological scores, grip strength and range of motion (ROM).

Measurement	Preoperatively	6 months postoperatively	12 months postoperatively
DASH (0–100 p)	45 (29–57)	32 (22–43)	29 (14–43)
PRWE (0–100 p)	64 (55–78)	48 (30–64)	48 (23–63)
PCS (0–52 p)	19 (12–27)	13 (7–21)	13 (5–21)
HADS depression (0–21 p)	2 (1–5)	2 (1–5)	3 (1–5)
HADS anxiety (0–21 p)	4 (2–7)	4 (1–7)	4 (1–7)
SOC (13–91 p)	73 (66–81)	74 (65–80)	75 (64–82)
EQ5D (0–1 p)	0.69 (0.23–0.80)	0.73 (0.66–0.79)	0,73 (0.62–0.80)
Grip strength (kg)	22 (13)	—	24 (11)
ROM (flexion–extension arc) (°)	78 (26)	—	73 (29)

All PROMs and psychological scores are presented as median (IQR). Grip strength and ROM are presented as mean (SD).

DASH: Disabilities of the Arm, Shoulder, and Hand; PRWE: Patient-Rated Wrist Evaluation; PCS: Pain Catastrophizing Scale; HADS: Hospital Anxiety and Depression Scale; SOC: sense of coherence; EQ5D: EuroQol 5D-3L; ROM: range of motion; p: points.

Table 3 presents the results of the primary GEE analyses. DASH, PRWE and EQ5D improved significantly 6 and 12 months after surgery and all PROMs reached the MCID-levels of the respective scales (Sorensen et al., 2013; Walters and Brazier, 2005). Grip strength also improved, but there was no change in ROM.

**Table 3.** Effect of surgery on patient reported outcome measures, grip strength and range of motion.

Measurement	Preoperatively - 6 months beta coefficient (*p*-value)	Preoperatively - 12 months beta coefficient (*p*-value)
DASH (0–100)^a^	−10 (***p *< 0.001**)	−13 (***p *< 0.001**)
PRWE (0–100)^a^	−16 (***p *< 0.001**)	−21 (***p *< 0.001**)
EQ5D (0–1)^a^	0.12 (***p *< 0.001**)	0.13 (***p *< 0.001**)
Grip strength (kg)^a^	—	2.2 (***p *= 0.039**)
ROM (flexion–extension arc) (°)^a^	—	−5.8 (*p *= 0.13)

^a^Adjusted for time, age, sex, type of surgery, preoperative SOC ≥ 73, preoperative PCS ≥ 30 and preoperative HADS anxiety ≥ 8.

Beta coefficient: expected population mean change of the outcome variables between the assessments; DASH: Disabilities of the Arm Shoulder and Hand; PRWE: Patient-Rated Wrist Evaluation; EQ5D: EuroQol 5D-3L; ROM: range of movement.

Statistically significant *p*-values shown in bold.

Sixteen patients had a PCS score ≥30 at baseline. PCS ≥ 30 had a negative predictive effect on both DASH (β = 10; *p *= 0.007), PRWE (β = 9; *p *= 0.032) and grip strength (β = −5; *p *= 0.023), but no effect on EQ5D or ROM.

HADS depression was omitted from the analyses since only five patients had a score of ≥8 at baseline. Nineteen patients had a HADS anxiety score of ≥8. A preoperative HADS anxiety score ≥8 had a negative predictive effect on DASH (β = 14; *p *= 0.002), PRWE (β = 14; *p *= 0.002) and EQ5D (β = −0.19; *p *< 0.001) at follow-up. A baseline HADS anxiety score ≥8 did not influence grip strength or ROM.

A high SOC did not have a significant impact on any of the outcomes. Partial wrist denervation had a small but significant negative predictive effect on PRWE (β = 8; *p* = 0.017) and a positive predictive effect on ROM compared with partial wrist fusion (β = 17; *p* < 0.001).

Higher age had a negative predicted effect on DASH, PRWE, EQ5D, grip strength and ROM at follow-up (β = 0.45 (*p *< 0.001); β = 0.41 (*p *< 0.001); β = –0.003 (*p *= 0.015); β = −0.39 (*p *< 0.001); and β = –0.44 (*p *= 0.004)) for every 1-year increase in age). Female gender predicted a worse DASH score (β = 9; *p *= 0.031) and a lower grip strength (β = −14; *p *< 0.001).

There was a significant improvement of PCS 6 months after surgery and PCS improved even more by 12 months (Table 4). The HADS anxiety and depression scores and SOC were stable during the study period with no significant changes over time. Age, sex and type of surgery did not affect the changes of the psychological scores.

**Table 4.** Changes in psychological scores after surgery.

Measurement	Preoperatively – 6 months beta coefficient (*p*-value)	Preoperatively – 12 months beta coefficient (*p*-value)
PCS^a^	–3.9 (***p *< 0.001**)	–5.0 (***p *< 0.001**)
HADS depression^a^	0.25 (*p *= 0.38)	0.23 (*p *= 0.40)
HADS anxiety^a^	–0.28 (*p *= 0.34)	–0.25 (*p *= 0.31)
SOC^a^	–0.26 (*p *= 0.83)	0.18 (*p *= 0.88)

^a^Corrected for time, age, sex and type of surgery. Beta coefficient: expected population mean change of the outcome variables between the assessments.

PCS: pain catastrophizing scale; HADS: hospital anxiety and depression scale; SOC: sense of coherence.

Statistically significant *p*-values shown in bold.

## Discussion

In patients with painful wrist osteoarthritis, we found that pain catastrophizing or a tendency for anxiety had a negative impact on the patient-reported outcomes after salvage procedures. Sense of coherence did not influence outcome.

Others have reported negative effects of pain catastrophizing and anxiety on PROMs after surgery for various chronic conditions and injuries to the hand and wrist (Egloff et al., 2017; London et al., 2014; Mosegaard et al., 2020; Ryan et al., 2022, Vranceanu et al., 2014). Similar results have also been reported in other fields of surgery, such as general, thoracic and cardiac surgery (Levett and Grimmett, 2019). Comparison of the levels of negative impact of psychological factors is complicated, because of differences in reported outcomes, statistical methods and whether one or several psychological factors are included in the analyses. Altogether, our results add to the increasing body of evidence on the negative influence of pain catastrophizing and anxiety on postoperative outcomes.

In our sample, pain catastrophizing had a negative impact on grip strength but not on ROM, whereas anxiety did not influence grip strength or ROM. Pain catastrophizing has been shown to have a negative effect on ROM after surgery for distal radial fractures (Teunis et al., 2015). Although these results after an acute injury are not entirely comparable with our patients who were recovering after salvage surgery, the findings imply that pain catastrophizing may delay or permanently worsen recovery of objective function after hand surgery, possibly because of apprehension about pain during mobilization.

We report that anxiety, but not pain catastrophizing, has a strong negative impact on quality of life. The negative association between anxiety and quality of life has been confirmed by a recent systematic review (Hohls et al., 2021) whereas a study on knee replacement surgery has suggested a correlation between pain catastrophizing and a lower EQ-5D (Birch et al., 2019).

There is a need for studies on psychological factors with a positive effect on outcomes (MacDermid et al., 2018). Satisfaction with life and mindfulness have been associated with less pain in upper extremity musculoskeletal disorders (Beks et al., 2018; Talaei-Khoei et al., 2018) and a high SOC has been correlated to better perceived general health (Eriksson and Lindström, 2006). Conversely, a low SOC has been associated with worse cold sensitivity after traumatic hand injuries and in hand-arm vibration syndrome (Carlsson and Dahlin, 2014) and a worsened DASH after major hand injury (Cederlund et al., 2010). Furthermore, a low SOC predicted a generally worse outcome after revascularization and replantation (Rosberg, 2014). Hence, we expected that a high SOC would predict a better outcome, but we found no effect of a high SOC on PROMs, grip strength or ROM. The differences in results may reflect differences in study designs and that we investigated a chronic condition, whereas others have studied acute hand traumas.

We found that anxiety and SOC were stable over time, whereas pain catastrophizing diminished during the year after surgery. In accordance with this, reduction of PCS has been reported after total knee arthroplasty (Høvik et al., 2016) and after rehabilitation for proximal or distal radial fractures (Golkari et al. 2015). There is additional support that pain catastrophizing may be modifiable by patient education and other inventions (Gibson and Sabo, 2018). Hence, pain catastrophizing may not be a fixed mindset, but potentially improvable by preoperative interventions and by an effective surgical treatment. To our knowledge, no previous studies have investigated whether HADS is constant over time or could be altered by surgery. Our finding that SOC is stable over time, is in line with a previous report on patients undergoing rehabilitation for various hand-related disorders, such as arthrosis, fractures and injuries to tendons, ligaments or nerves (Hansen et al., 2017).

The major limitation of this study is the lack of control group, with a resultant risk for bias and confounding. Demographic data are included in the analysis to reduce the risk of confounding, but the risk of bias cannot be accounted for. The study population consisted of patients that had either a partial wrist denervation or a midcarpal fusion, which is also a potential weakness. To control for any differences in outcomes attributable to the surgical treatments, the type of surgery was included as a covariate in the GEE analyses. Depression has been reported to influence postoperative outcome in hand and wrist surgery (Crijns et al., 2019; Vranceanu et al., 2010; Yeoh et al., 2016). Owing to the very low number of patients with a suspected depression in our sample, we did not include depression in the analyses. A larger sample size is needed to establish whether depression affects postoperative outcome in wrist osteoarthritis.

The foremost strengths of this study are the prospective longitudinal design with the loss of few patients and the pre- and postoperative assessments by validated and widely used upper-extremity and wrist-specific PROMs and well-established psychological scores.

In summary, we found a negative predictive effect of pain catastrophizing and anxiety on patient-reported outcomes after salvage surgery for wrist osteoarthritis. Anxiety also predicted a lower postoperative quality of life, whereas pain catastrophizing had a negative impact on grip strength. Anxiety and depressive status seem to be stable over time, whereas pain catastrophizing may be improved by the surgical treatment itself. Psychological problems are common and hand surgeons need to be aware of the negative impact of preoperative psychological problems on the outcomes of surgery. The outcomes may be improved by identifying patients with these conditions, and by giving them preoperative counselling or treatment.
